# Rbec: a tool for analysis of amplicon sequencing data from synthetic microbial communities

**DOI:** 10.1038/s43705-021-00077-1

**Published:** 2021-12-06

**Authors:** Pengfan Zhang, Stjin Spaepen, Yang Bai, Stephane Hacquard, Ruben Garrido-Oter

**Affiliations:** 1 Department of Plant-Microbe Interactions, Max Planck Institute for Plant Breeding Research, 50829 Cologne, Germany; 2 CMPG Laboratory of Genetics and Genomics, Department M2S, KU Leuven, Gaston Geenslaan 1, 3001 Leuven, Belgium; 3 State Key Laboratory of Plant Genomics, Institute of Genetics and Developmental Biology, Innovation Academy for Seed Design, Chinese Academy of Sciences, 100101 Beijing, China; 4 Cluster of Excellence on Plant Sciences (CEPLAS), Max Planck Institute for Plant Breeding Research, Cologne, Germany

**Keywords:** Microbial communities, Microbial ecology

## Abstract

Synthetic microbial communities (SynComs) constitute an emerging and powerful tool in biological, biomedical, and biotechnological research. Despite recent advances in algorithms for the analysis of culture-independent amplicon sequencing data from microbial communities, there is a lack of tools specifically designed for analyzing SynCom data, where reference sequences for each strain are available. Here we present Rbec, a tool designed for the analysis of SynCom data that accurately corrects PCR and sequencing errors in amplicon sequences and identifies intra-strain polymorphic variation. Extensive evaluation using mock bacterial and fungal communities show that our tool outperforms current methods for samples of varying complexity, diversity, and sequencing depth. Furthermore, Rbec also allows accurate detection of contaminants in SynCom experiments.

Amplicon sequencing is a powerful technique to characterize the composition of microbial communities from environmental samples. Recent advances in algorithms and tools for the analysis of marker gene amplicon data have driven a shift from clustering approaches, based on operational taxonomic units (OTUs) and arbitrary sequence similarity thresholds, to error correction methods [1–4] that seek to estimate abundances of individual amplicon sequence variants (ASVs). A new generation of integrated pipelines [5] allows researchers from a variety of fields in the environmental, biological, and medical sciences to reproducibly analyze marker gene sequencing data.

Synthetic microbial communities (SynComs) constitute an emerging and powerful tool to build experimentally tractable, reproducible microbial systems in the laboratory that enable controlled perturbation experiments and testing of falsifiable hypotheses. These bottom-up, reductionist approaches are being increasingly employed in studies of microbial ecology and evolution [6], plant and animal microbiota [7–9], and biotechnology [10]. A factor limiting these innovative experimental approaches from developing to their full potential is the lack of bioinformatic tools specifically designed for the analysis of sequencing data obtained from gnotobiotic systems and SynComs. As a result, researchers typically employ standard clustering, error correcting or mapping approaches that do not take full advantage of these tractable experimental systems (e.g., reduced community complexity and the availability of reference sequences for classification), resulting in reduced resolution, accuracy or data loss. To address this limitation, we developed a reference-based error correction algorithm that is able to accurately and precisely correct PCR and sequencing errors in SynCom amplicon data, identify intra-strain polymorphism, and detect the presence of contaminants in gnotobiotic systems.

Here, we introduce Rbec, an easy-to-use tool, freely available as an R package, that not only corrects amplicon sequencing errors by implementing a modified version of the quality-aware model implemented in the DADA2 tool [1], but also identifies intra-strain polymorphic variation and contaminants in samples of SynComs. Rbec is specifically designed to efficiently and accurately process data from SynComs, for which reference sequences of individual community members are available (Fig. 1). A detailed description of the Rbec algorithm is provided in the Supplementary Information and an overview is given below.

**Fig. 1:**
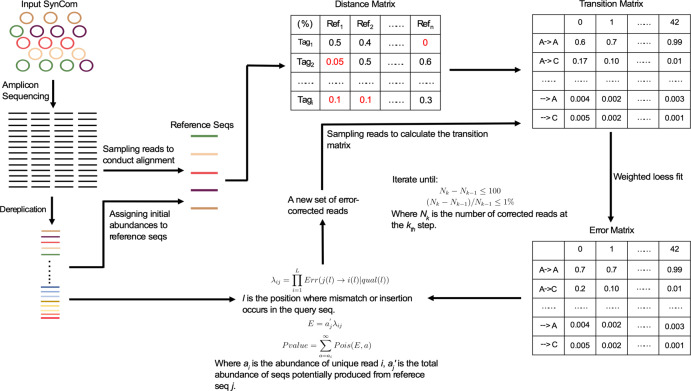
Schematic diagram of the Rbec algorithm. Rbec consists of two main steps: error matrix estimation and abundance probability calculation. For the error matrix estimation, Rbec traverses through all query reads and reference sequences and matches each read with a unique candidate error-producing reference. Alignments between input and reference sequences are then used to calculate the error matrix. Finally, abundance probabilities are estimated by fitting a *Poisson* distribution.

First, reads are de-replicated into unique tags and subsequently aligned to the reference database containing amplicon sequences from SynCom members, typically generated from sequencing of clonal cultures. Initial abundances are then assigned to each strain according to the copy number of each exactly aligned tag. Next, tags that are not exactly matched to any sequence in the database are assigned a candidate error-producing reference based on *k*-mer distances. Sequencing reads are then subsampled and an error matrix is calculated using the mapping between subsampled reads and candidate error-producing sequences. The probability that a unique tag is erroneously produced by a given candidate error-producing sequence is then calculated using a Poisson distribution. The probability and expectation values of this distribution are then used to determine whether a unique tag can be corrected from a reference sequence, or whether it can be identified as originating from a paralogous sequence. Tags that cannot be corrected are subsequently removed. The parameters of the error model are recomputed iteratively until the number of re-assignments falls below a set threshold. Strain abundances are then estimated from the number of error-corrected reads mapped to each reference sequence. Finally, potentially contaminated samples are identified by assessing a significant deviation from the expected proportion of corrected reads. Sequences of putative contaminants then provided as an output for further examination.

To validate the performance of Rbec, we employed mock samples generated using a taxonomically wide set of 236 bacterial and 97 fungal strains obtained from sequenced culture collections derived from the *Arabidopsis thaliana* microbiota [7, 11]. Mapping of amplicon reads to the reference sequences showed that only 31.8% of all reads per sample, on average, had a perfect match in the database, indicating the presence of extensive sequencing and PCR errors and polymorphic copies (Fig. 2A, and Supplementary Fig. S1). Our implementation of the Rbec algorithm successfully corrected most erroneous reads (89.2% on average), out-performing all other tested de novo correction methods (Fig. 2B). This improvement was most pronounced for reads generated from polymorphic copies of marker sequences within a single strain, owing to the fact that Rbec is capable of correctly classifying paralogous sequences (Supplementary Fig. S2).

**Fig. 2:**
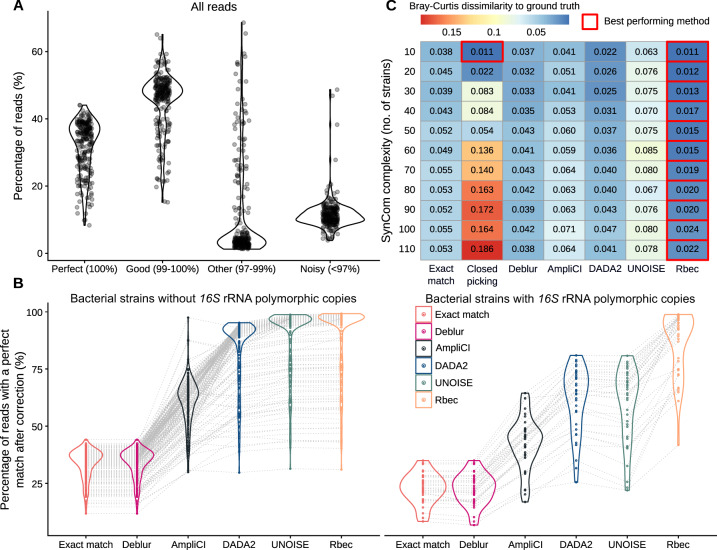
Evaluation of the Rbec algorithm. **A** Error profiles of amplicon sequencing data from 234 bacterial strains sequenced on the Illumina platform. **B** Percentage of perfectly aligned reads after correction with different methods, including samples generated from strains with or without polymorphic copies of the 16S rRNA gene. **C** Evaluation of the influence of community complexity on the performance of different methods, measured as a deviation from the ground truth using Bray-Curtis dissimilarities. The columns represent the different methods and the rows correspond to the number of strains used to generate the SynCom mock community data. The values inside the heatmap refer to the averaged Bray-Curtis dissimilarities over 20 replicates for each SynCom combination.

To evaluate the accuracy of Rbec in characterizing community composition, we simulated in silico bacterial and fungal mock samples by mixing reads generated from sequencing individual isolates separately (Supplementary Fig. S3). For these simulations, we varied community complexity, strain similarity and sequencing depth. Across these three parameters, Rbec consistently performed better than all other tested methods in characterizing microbial composition in terms of deviation from the ground truth (Fig. 2C, Supplementary Figs. S4 and S5), as well as precision and recall (Supplementary Fig. S6), while robustly being able to identify contaminated samples (Supplementary Fig. S7).

Rbec is easy to use and highly customizable. Despite being parallelizable, it can also be run in a standard modern desktop or laptop computer and process amplicon samples containing thousands of sequencing reads within minutes using a single CPU core (Supplementary Table S1). Rbec is freely available as an open-source multi-platform R package. Release versions can be obtained via Bioconductor. The developer version is maintained and can be downloaded at: https://github.com/PengfanZhang/Rbec.

## Supplementary information

Supplementary Information
