# Supplementary material for: Rbec: a tool for analysis of amplicon sequencing data from synthetic microbial communities
Source: ISME Commun. 2021 Dec 6;1:73. doi: 10.1038/s43705-021-00077-1 (PMC9723543; doi:10.1038/s43705-021-00077-1)
Supplement: Supplementary file 1 — Supplementary Information [file 43705_2021_77_MOESM1_ESM.pdf]

Rbec: a tool for analysis of amplicon sequencing data from synthetic microbial communities

Pengfan Zhang[1], Stjin Spaepen[3], Yang Bai[4], Stephane Hacquard[1,2], Ruben Garrido-Oter[1,2,*]

1 Department of Plant-Microbe Interactions, Max Planck Institute for Plant Breeding Research, 50829 Cologne, Germany.

2 Cluster of Excellence on Plant Sciences (CEPLAS), Max Planck Institute for Plant Breeding Research, Cologne, Germany.

3 CMPG Laboratory of Genetics and Genomics, Department M2S, KU Leuven, Gaston Geenslaan 1, 3001, Leuven, Belgium.

4 State Key Laboratory of Plant Genomics, Institute of Genetics and Developmental Biology, Innovation Academy for Seed Design, Chinese Academy of Sciences, 100101 Beijing, China.

*Correspondence to: garridoo@mpipz.mpg.de

Appendix

Detailed description of Rbec algorithm
Detection of contamination
Construction of accurate reference sequence databases
Differences with respect to other error-correction algorithms
Simulation of mock communities
Data processing with different methods
Time performance
Description of the output files
Description of the parameters
Library construction and preparation
Data deposition
Supplementary Figures
Supplementary Tables
References

Detailed description of the Rbec algorithm

A schematic workflow of Rbec is shown in **Fig. 1**. A detailed, step-by-step description of the algorithm is detailed below.

Dereplication and assignment of initial abundances

Initial abundances for each sequence present in the reference database are inferred on the basis of the number of exact matches found in the uncorrected sequencing reads. First, merged reads are dereplicated into unique tags in a sample-wise manner. Sequence quality scores for each unique tag are averaged over the scores of all identical copies of that sequence and for every residue. Each reference sequence is assigned an initial abundance equal to the number of identical copies of that unique tag found in the sample. If a reference sequence does not have any tags that exactly matches it, the strain from which the reference sequence is derived is marked as 'absent' from the sample.

Assignment of erroneous reads to error-generating reference sequences

Unique tags that do not exactly match any sequence in the reference database are initially assumed to originate from erroneous sequencing reads generated by a given reference sequence. In order to identify the most likely error-generating reference sequence for each unique tag, *k*-mer (k=7) distances between each unique tag and each reference sequence are calculated. We use *k*-mer distances for pairwise comparisons between unique tag queries and reference sequences instead of computationally intensive global alignments to improve the time performance of the algorithm. In addition, these distance calculations can be performed in parallel to further increase performance (see parameter 'threads'). The reference sequence showing the lowest *k*-mer distance to the query unique tag is marked as the candidate error-producing sequence from which the corresponding erroneous sequences originate. If multiple candidates with the same *k*-mer distance are found, only

the reference sequence with the highest initial abundance is considered as the original error-generating sequence, as sequences with higher abundances are more likely to generate erroneous sequences.

Estimation of the transition and error matrices

To calculate the probability that an unique tag is produced by a given error-generating reference sequence, transition and error matrices need to be estimated. The transition matrix is a 20 by 43 matrix where the rows represent the transition combinations (e.g., A→A, A→T, A→G, A→C, T→T, …, C→G, C→C; including insertions), and the columns represent the sequence quality scores. This transition matrix can be estimated by performing a global alignment between a random set of subsampled unique sequences (or 'tags'; 5,000 reads by default) and the reference sequences. Entries in the transition matrix are calculated by counting the number of each transition combination along the length of the alignment. The log-transformed transition matrix is then fitted with a weighted loess function to generate the error matrix.

Calculation of error-generation probabilities

We assume that the mismatches between query and reference are generated independently, so the rate at which a unique tag *i* is produced from the error-generating reference *j*, designated λ, is calculated by the product over the error probabilities at each position of the alignment *l*:

$$\lambda_{ij} = \prod_{l=1}^{L} Err(j(l) \rightarrow i(l) | qual(l))$$

Where *L* is the total length of the alignment.

Similar to the error-aware model implemented in DADA2 [1], the abundance probability of each unique tag is calculated using the *Poisson* distribution:

$$E = a_j^{'}\lambda_{ij}$$

$$Pvalue = \sum_{a=a_i}^{\infty} Pois(E, a)$$

Where E is the expectation of the *Poisson* distribution, a_i is the abundance of a unique tag i, and a_j' is the aggregated abundances of all unique tags assigned to a reference sequence j.

Unique tags with a *P*-value lower than 10^{-40}, and an expectation lower than 0.05 are discarded. This expectation cut-off is intended to retain tags that could be produced at least once by the reference with the probability above 5%. The aim of this step is to retain tags that are generated from intra-strain amplicon sequence variants, which show high abundance relative to the reference sequence but do not exceed the *P*-value cut-off. It is possible that, for certain experiments, modifying these parameters could be useful. For instance, in a community containing very low abundance strains, lowering the minimum expectation threshold might increase the sensitivity of the algorithm. Similarly, if the presence of low-abundance contaminants closely related to a reference strain is of particular concern, increasing the minimum *P*-value threshold will help identify potential contaminants, at the risk of generating a larger number of false positives.

As discussed above, Rbec is able to identify polymorphic paralogues of the marker gene for a given strain. By summing up the number of (polymorphic) paralogues identified for each reference sequence from each strain, we can obtain an estimate of the copy number of the marker gene for each strain in the SynCom. Normalization of the output relative abundances using this internally inferred copy number estimate is conducted by Rbec by default (see also documentation regarding output files), although this can be controlled by the user (see parameter ‘cn’). However, identical paralogues of the marker gene will not be detected, and the copy number inferred by Rbec may be

underestimated. Additionally, the user can provide a table with copy number information for each strain (e.g., obtained from whole-genome sequences, if available) for abundance normalization.

Iterative correction of unique tags

Tags above the *P*-value or *E* threshold are then randomly subsampled (5 000 reads by default) and aligned to the reference sequences in an iterative process. In each iteration, the error matrix is updated with tags corrected during the last iteration. The iterations continue until the number of corrected reads falls below two fixed thresholds, which we set to determine whether the iteration should stop or not. These two thresholds correspond to the absolute and relative differences in the number of corrected reads between the present and previous iterations, and are calculated as follows:

$$N_k - N_{k-1} \leqslant 100$$

$$(N_k - N_{k-1})/N_{k-1} \leqslant 1\%$$

N_k and N_{k-1} denote the number of corrected reads in the k_{th} and $k\text{-}1_{th}$ iteration respectively. The threshold based on relative differences is used to appropriately stop the iterative process for samples with low sequencing depths, since they can easily satisfy the cut-off based on absolute differences. Once both of these two conditions are met, iterations stop and each reference sequence is assigned an abundance equal to the aggregated abundance of all its assigned unique tags.

Detection of contamination

Existing error-correction algorithms designed for culture-independent community profiling data cannot accurately estimate the abundances of strains with marker gene paralogs, and show a strong bias towards underestimation of their abundances. In addition, paralog sequences are typically classified as sequence variants originating from different strains, resulting in an inflation of alpha-

diversity (within sample diversity). When amplicon sequencing data obtained from synthetic communities is analysed using approaches such as closed OTU-picking, reads from paralog sequences are discarded, leading to low percentages of aligned reads per sample. Samples with high abundance of strains containing polymorphic paralogous marker sequences can thus be erroneously considered contaminated. Given that Rbec not only corrects most erroneous amplicon sequencing reads, but also successfully identifies paralogous sequences, we can assume that a high proportion of uncorrected reads is likely the result of contamination.

We evaluated the capacity of Rbec to identify contaminated samples by performing an *in silico* simulated dataset, where we included amplicon sequences from *Escherichia coli* with 5% relative abundance per 'contaminated' mock sample. When examining the percentage of reads successfully corrected per sample (**Fig. S6**), we observed a clear separation between 'clean' and 'contaminated' mock samples. Based on these results, we included a function in Rbec that can be used to flag potentially contaminated samples and output the amplicon sequences of putative contaminants.

A sample i is flagged as contaminated, if

$$R_i < \mu - 1.5IQR$$

Where R_i is the recruitment ratio of reads of sample i, μ is the mean of recruitment ratio of reads across all samples in the dataset, and IQR is the interquartile range of the recruitment ratio. If a sequence accounts for more than 3% of total reads after error correction, we assume this sequence originates from a contaminant strain. The accuracy of this heuristic approach depends on the abundance of the contaminant as well as in its prevalence across samples within a dataset. When contamination occurs in the majority of the samples in a dataset, low read requitement rations would be observed in general, and no individual samples will be flagged. Based on our analyses,

samples with less than 90% input reads successfully corrected should be considered as potentially contaminated and further examined. If low recruitment ratios are observed across all samples in dataset, putative contaminant sequences provided by Rbec, which can then be used for further analysis. If no prevalent contaminant sequences are identified across samples, low percentages of error-corrected reads can be attributed to other technical factors, such as derelict DNA from the environment or unusually high PCR or sequencing errors.

Construction of accurate reference sequence databases

Analysis of amplicon data derived from SynCom experiments relies on the use of accurate reference sequences derived from each strain. As explained above, Rbec uses this reference database for error correction, identification of polymorphic paralogs, and contaminant detection. Generally, these reference sequences have been previously generated from clonal cultures of individual SynCom members, either by extraction from their respected whole-genome sequences or by Sanger sequencing of the specific marker sequence.

Independently of the method, inaccuracies in the references are likely to occur. For instance, assembly and subsequent extraction of rRNA gene sequences from genomes obtained using short-read technologies are common, and PCR or sequencing errors from targeted amplification could likewise result in erroneous sequences. Whenever errors are present in the reference sequence, no identical unique tags will be identified during the first step of the Rbec algorithm, and the corresponding strain will be labeled as 'absent' from all samples. Other methods, such as closed OTU picking suffer from similar pitfalls, making this a general problem for SynCom data analysis.

However, errors in the reference database can be corrected by leveraging the feature described above, which allows Rbec to predict potential contaminants. If a reference sequence is inaccurate but its corresponding strain is found in a given SynCom sample with at least a 3% relative

abundance, the ratio of corrected reads will decrease and Rbec will output the correct sequence as a putative contaminant. By identifying contaminant sequences with high similarity to reference sequences from strains labeled as 'absent', these inaccuracies can be readily corrected. Once the reference database has been updated, Rbec can be re-run on the same dataset to obtain accurate strain abundances.

Differences with respect to other error-correction algorithms

Current error-correcting methods for amplicon sequencing data (e.g., DADA2, Unoise, Deblur, and AmpliCI) are designed for the analysis of natural communities, for which we have limited prior knowledge about the microbial strains present in a sample. These algorithms identify the amplicon variants present in a sample by comparing low-abundance to high-abundance sequences. Rbec, however, is designed for processing SynCom data, and utilizes prior knowledge regarding community composition, which is provided by the user as a nucleotide FASTA file containing reference amplicon sequences for SynCom members. Several differences account for the improved performance of Rbec when analyzing SynCom data. Firstly, Rbec can more accurately identify sequences from low-abundance strains which are included in the reference database and which other error-correcting methods might falsely classify as errors. In addition, Rbec is able to detect the presence of polymorphic paralogues of the amplified marker sequence within the same strain. This is accomplished by implementing a threshold based on the expectation of the *Poisson* distribution, relying on the fact that polymorphic paralogues will have a high expectation value due to their similarity to the reference sequence of the strain from which they originate. Finally, Rbec takes into account insertions when generating the error matrix, unlike other algorithms such as DADA2.

Simulation of mock communities

To evaluate the performance of the different algorithms when analysing with SynCom data with varying complexities, strain similarities, and sequencing depths, we simulated mock samples using data obtained from sequencing of clonal cultures individually. Firstly, reference sequences of the V5-V7 region from all the bacterial strains were dereplicated, resulting in 114 strains having unique sequences in the V5-V7 region. We first evaluated the performance of different methods by analysing samples obtained from single strain cultures (Fig. 1). To assess the impact of the presence of polymorphic copies of the marker gene, we differentiated samples derived from bacterial strains with or without *16S* rRNA polymorphism using information from their whole-genome assemblies. Next, we generated mock community samples with different complexities, 10 to 110 strains from the candidate list containing 114 strains were randomly picked for each mock sample, with a step size of 10 strains. The relative abundance of each strain was simulated using a log normal distribution (s.d. = 2). The total number reads in each mock sample was fixed at 10 000 reads, and the reads for each strain were subsampled from the amplicon sequencing output of each individual strain using Seqkit [2]. For instance, to generate a mock community with 3 strains, with relative abundances of 70%, 20%, and 10% respectively, 7 000, 2 000, and 1 000 reads would be sampled from each of the three individual amplicon samples and subsequently mixed to generate a mock sample (**Fig. S7**).

To generate mock samples with different strain similarities, we set a maximum pairwise similarity threshold between each pair of strains in each mock community, ranging from 85% to 100%. To alleviate the influence of uneven abundance distribution of each strain on the evaluation, only 20 strains with equal abundances (5% relative abundance for each strain) were included in mock communities. Similarly, to evaluate the impact of different sequencing depths on the performance

of the different algorithms, we simulated mock data with 50 strains at different depths, ranging from 500 to 10 000 reads. For each evaluation category, we generated 20 replicates for each parameter combination.

In addition to mock samples of bacterial communities, we also generated fungal mock communities for the purpose of evaluating our algorithm on a different marker gene. 97 fungal strains with unique ITS1 sequences were set as seeds and randomly chosen to generate the fungal mock communities.

Data processing with different methods

Raw reads were merged using Flash2 [3] with parameters '-m 0.25 -M 250'. Merged reads with ambiguous bases were excluded with USEARCH [4]. DADA2 and Deblur plug-ins in QIIME2 [5] were applied to the filtered data by following the protocols indicated on the QIIME2 website (https://docs.qiime2.org/2019.7/tutorials/), to correct the reads and generate ASV tables. We also included the recently published algorithm AmpliCI [6] for comparison purposes. We followed the instructions on the corresponding Github website (https://github.com/DormanLab/AmpliCI) to process the data and generate the ASV table. The abundances of ASVs showing exact matches to the reference sequences were extracted from this feature table. For the UNOISE tool [7], filtered reads were dereplicated by USEARCH and subsequently denoised using the -unoise3 function in USEARCH. For the exact match method, the filtered sequencing reads were aligned to the reference database by running the -uparse_ref command in USEARCH. Only hits with 100% identities were retained for generating the profiling table. The Deblur and AmpliCI methods were not applied to fungal data since the two methods require the equal length of input data, while the length of ITS sequences shows a large variation among strains.

We applied the DADA2, UNOISE, Deblur, exact match, closed OTU picking, and Rbec methods to the simulated data sets. Finally, we calculated the Bray Curtis dissimilarities between the predicted profiling tables from different methods and the real composition for each mock sample (ground truth) using the *vegan* R package [8] to evaluate the performance of each algorithm. Significance differences between methods were assessed using a pairwise Wilcoxon test.

We also compared the precision and recall of the two second-best performing methods, namely, DADA2 and exact match classification with Rbec, using the following formulas:

$$Recall = \frac{No.\ of\ precisely\ corrected\ \text{reads}}{Total\ No.\ of\ reads}$$

$$Precision = \frac{No.of\ precisely\ corrected\ reads}{No.of\ corrected\ reads}$$

Time performance

The CPU time of Rbec on a single sample was tested on an Intel processor (Intel(R) Xeon(R) CPU E5-4657L v2 @ 2.40GHz) with 48 CPUs and 756 GB RAMs. Rbec can analyse 10 000 reads from a SynCom sample comprising of 100 strains within 3 minutes by using single CPU. The comparison of time performance among different methods can be found in **Table S1**. All the methods were tested on 5 simulated bacterial SynComs with containing 100 strains at a depth of 10 000 sequencing reads each by using 1 CPU.

Description of output files

- **strain_table.txt**: tab-separated file containing the strain-level community composition table.
- **strain_table_normalized.txt**: tab-separated file strain-level community composition table, normalized using copy-number information (see also documentation on the parameter ‘cn’).

- **contamination_seq.fna**: nucleotide FASTA file containing the sequences of potential contaminants.
- **rbec.log**: text file containing the percentage of corrected reads per sample, which can be used to predict potentially contaminated (see also parameter 'min_cont_abs').
- **paralogue_seq.fna**: nucleotide FASTA file including sequences of polymorphic paralogues sequences found for each strain.
- **lambda_final.out**: test file containing the lambda and *P*-values of the *Poisson* distribution for each unique tag.
- **error_matrix_final.out**: text file containing the error matrix used in the final iteration.

Description of parameters

- **fastq**: path of the FASTQ file containing the merged amplicon sequencing reads (Ns are not allowed in the reads).
- **reference**: path to the nucleotide FASTA file containing the unique reference sequences. Each sequence must be in one line (Ns are not allowed).
- **outdir**: path to the output directory.
- **threads**: number of threads used, default 1.
- **sampling_size**: sampling size for calculating the error matrix, default 5,000.
- **ascii**: ASCII characters used to encode phred scores (33 or 64), default 33.
- **min_cont_obs_abd**: minimum observed abundance for a non-corrected unique tag to be considered a potential contaminant (default 200).
- **min_cont_abd**: minimum relative abundance for a non-corrected unique tag to be considered a potential contaminant (default 0.03).

- **min_E**: minimum value expectation of the *Poisson* distribution for identification of paralogues (default 0.05).
- **min_P**: minimum *P*-value of the *Poisson* distribution required to correct a sequencing read (default 1e-40).
- **ref_seeker**: method used for finding the candidate error-producing reference sequence for a tag showing identical lowest *k*-mer distance to multiple references. 1 for the abundance-based method; 2 for the transition probability-based method, default 1.
- **cn**: path to a tab-separated table containing the copy number of the marker gene for each strain. The first column of the table should contain the strain IDs, and the second the copy number estimates (real values are allowed). If no table is provided, Rbec will normalize the abundance based on the internally inferred copy number (which tends to be un underestimate). Rbec will output the normalized as well as the non-normalized abundance tables. Default NULL.

Sequencing library construction and preparation

To evaluate the errors in the output from sequencing and test this algorithm, we performed amplicon sequencing on clonal cultures of 236 individual bacterial strains and 97 fungal strains isolated from the root of *Arabidopsis thaliana* separately [9, 10]. Genomic DNA was isolated from each strain using the MP Biomedicals FastDNATM Spin Kit for Soil. DNA concentration was determined fluorometrically using the Quant-iT PicoGreen dsDNA Assay Kit (Thermo Fisher Scientific). The V5-V7 region of the *16S* rRNA gene in bacteria and ITS1 region in fungi was amplified using the AACMGGATTAGATACCCKG (799F) and ACGTCATCCCCACCTTCC (1192R) primers, and CTTGGTCATTTAGAGGAAGTAA (ITS1F) and GCTGCGTTCTTCATCGATGC (ITS2R) primers, respectively. Indexing was done using

Illumina-barcoded primers. The indexed amplicons were subsequently pooled, purified, and sequenced on the Illumina MiSeq platform.

To exclude the possibility that the observed error distribution of amplicon sequencing is specific to the MiSeq platform, we also analysed the amplicon sequencing data obtained using a HiSeq platform [11] (**Fig. S1**).

Data deposition

Raw sequencing data used to generate mock bacterial and fungal community data were deposited into the European Nucleotide Archive (ENA) under the accession number PRJEB43511. The scripts used for the computational analyses described in this study are available at https://github.com/PengfanZhang/Rbec, to ensure replicability and reproducibility of these results.

Supplementary Figures

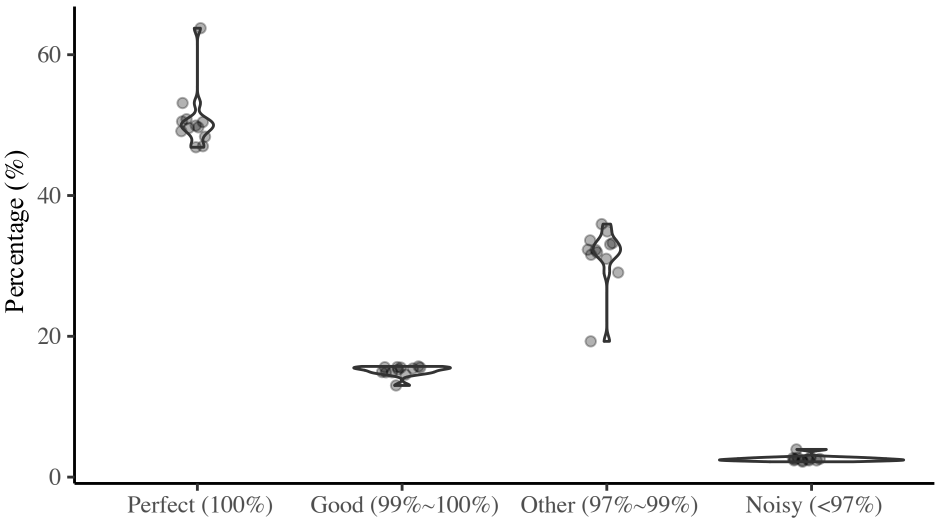

Figure S1. Error distribution of amplicon sequencing data from a HiSeq sequencer. Error profiles of amplicon sequencing data from 12 SynCom samples comprised of 12 bacterial strains and an artificial spike-in plasmid sequenced using the Illumina HiSeq platform.

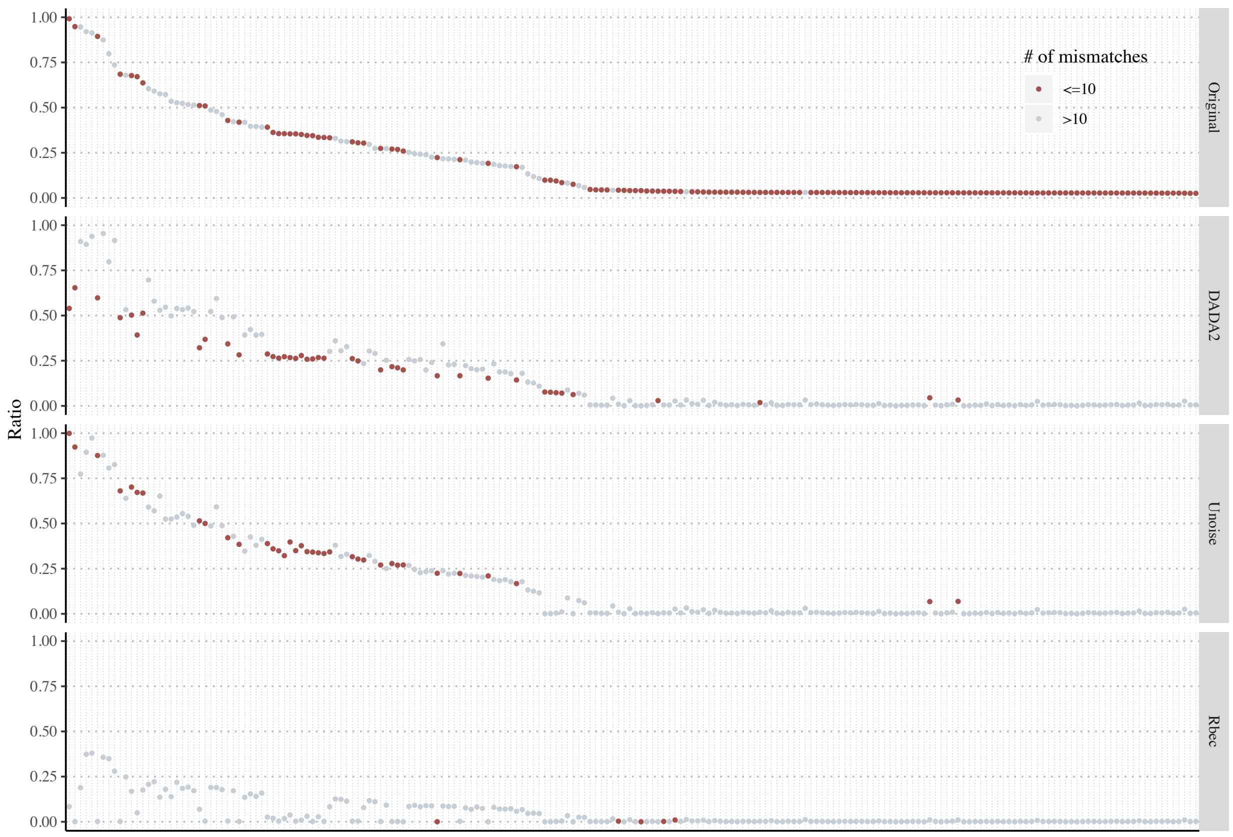

Figure S2. Abundance ratios between second-most abundant and most abundant unique tags in each sample before and after correction with different methods. The x axis represents different strains and y axis represents the abundance ratio calculated as $\frac{Abundance\ of\ second-most\ abundant\ unique\ tag}{Abundance\ of\ most\ abundant\ unique\ tag}$. The colour of each dot indicates the sequence dissimilarity between the two unique tags. Data points are depicted in dark brown if the two tags are close (≤ 10 base mismatches).

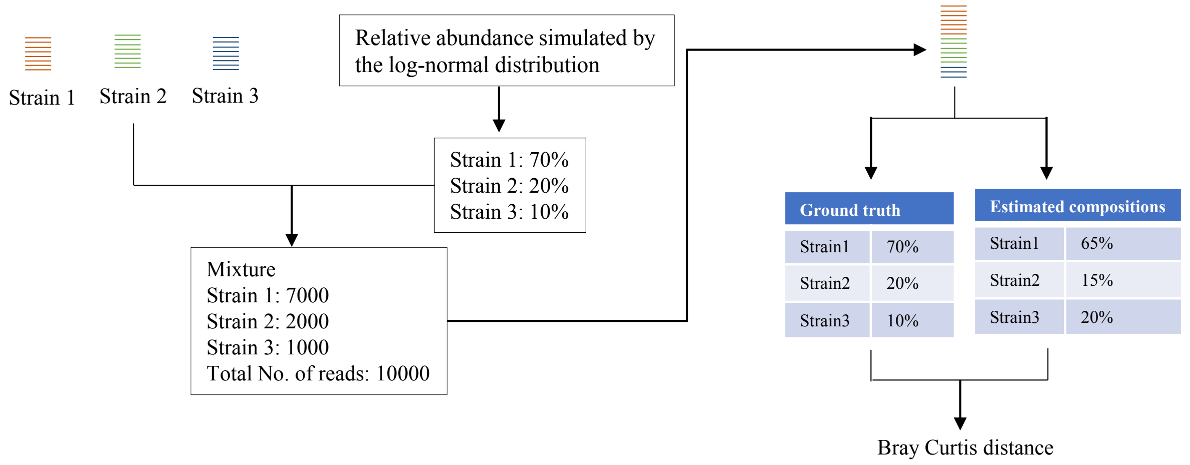

Figure S3. Workflow for the generation of mock dataset sand performance evaluation. Example of the *in silico* generation of a dataset from a mock community consisting of 3 strains. The relative abundance of each strain is simulated by a log-normal distribution. amplicon reads from each strain are subsampled from amplicon samples sequenced from clonal cultures and mixed according to their relative abundance. For evaluation of the different methods, Bray Curtis dissimilarities between the simulated relative abundances (ground truth) and the estimated compositions obtained by different methods are calculated.

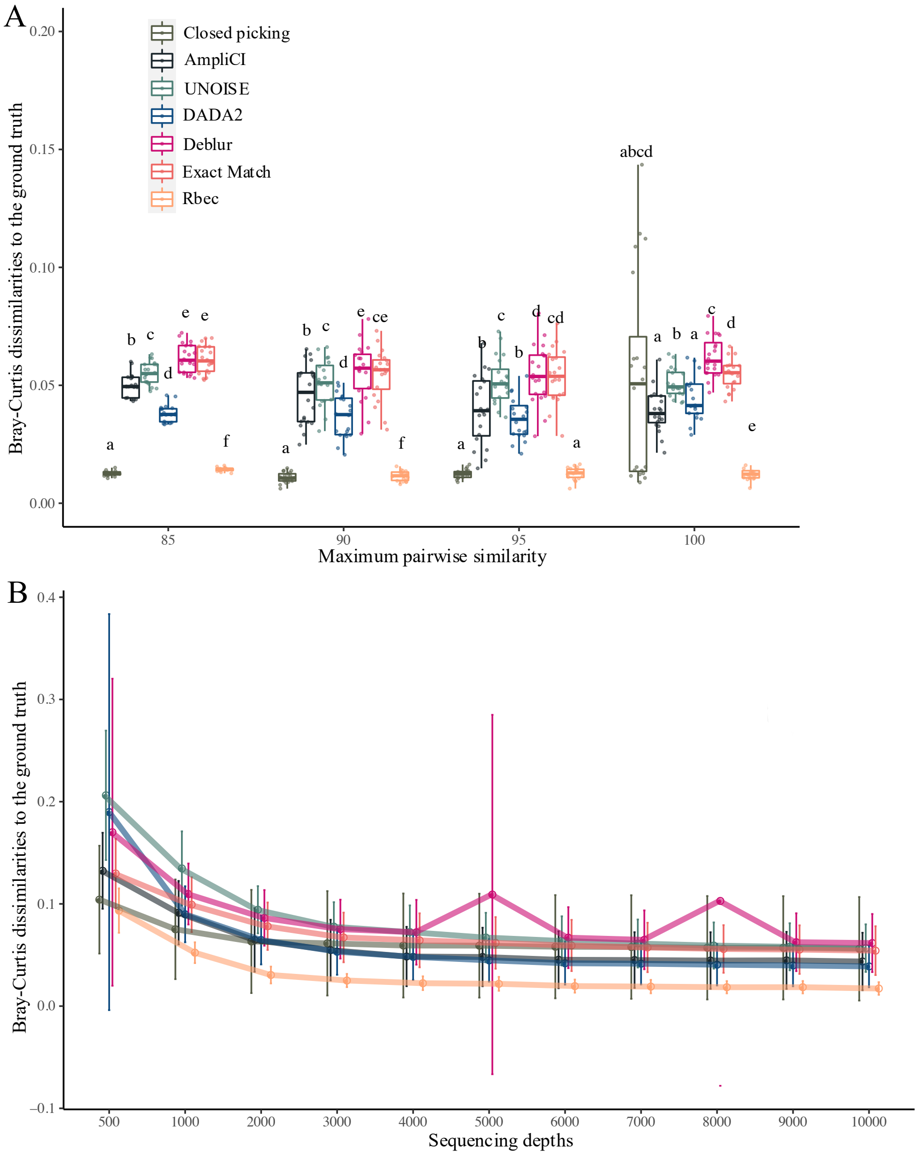

Figure S4. Effect of strain relatedness (A) and sequencing depth (B) on the performance of different methods to characterize bacterial communities. (**A**) Mock samples were generated from 20 strains with different strain similarities and 20 replicates for each threshold. Letters indicate the significant groups (paired Wilcox test, $P < 0.05$) within mocks with the same strain similarity. (**B**) The mock samples with different sequencing depth were directly subsampled from the mock samples with 50 strains. Circles represent the means of each method and parameter combination, while vertical lines represent the standard deviation.

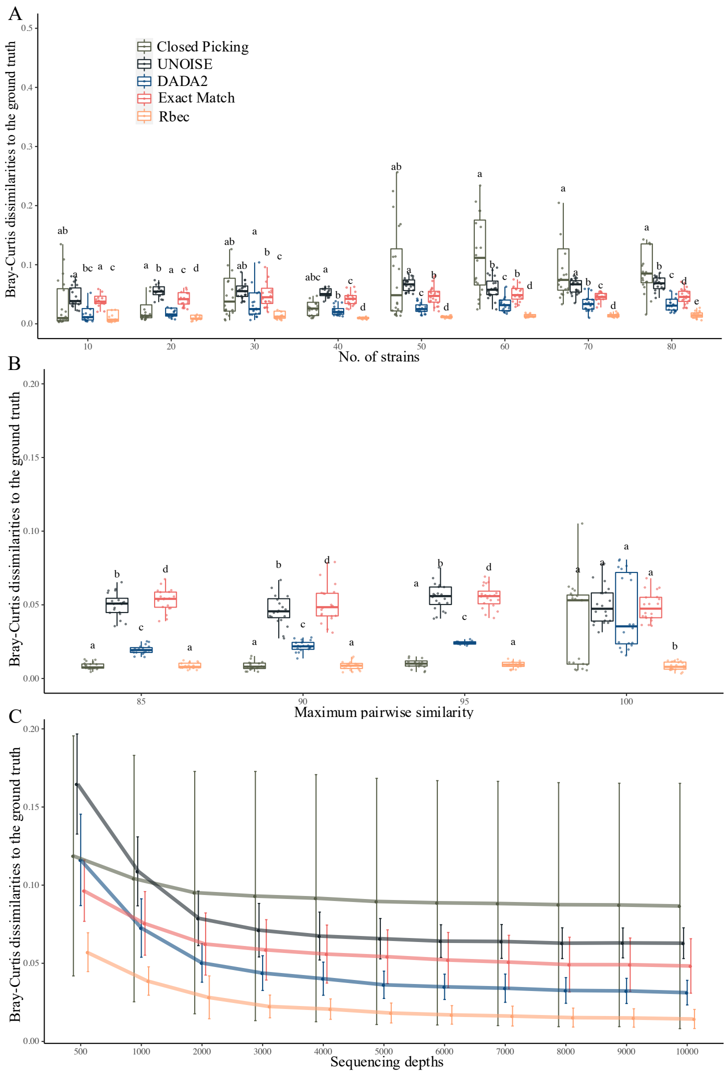

Figure S5. Effect of community complexity (A), strain relatedness (B) and sequencing depth (C) on the performance of different methods to characterize fungal communities. Mock samples were generated by randomly picking fungal strains from 97 candidates and mixing subsampled reads from the corresponding strains. For mock samples with different similarities, only 20 fungal strains were included for each mock, while mock samples with different sequencing depths were directly subsampled from the dataset containing 50 fungal strains. For each threshold, 20 replicates were generated. Letters indicate the significant groups (paired Wilcox test, $P < 0.05$) within mock samples with the same parameters.

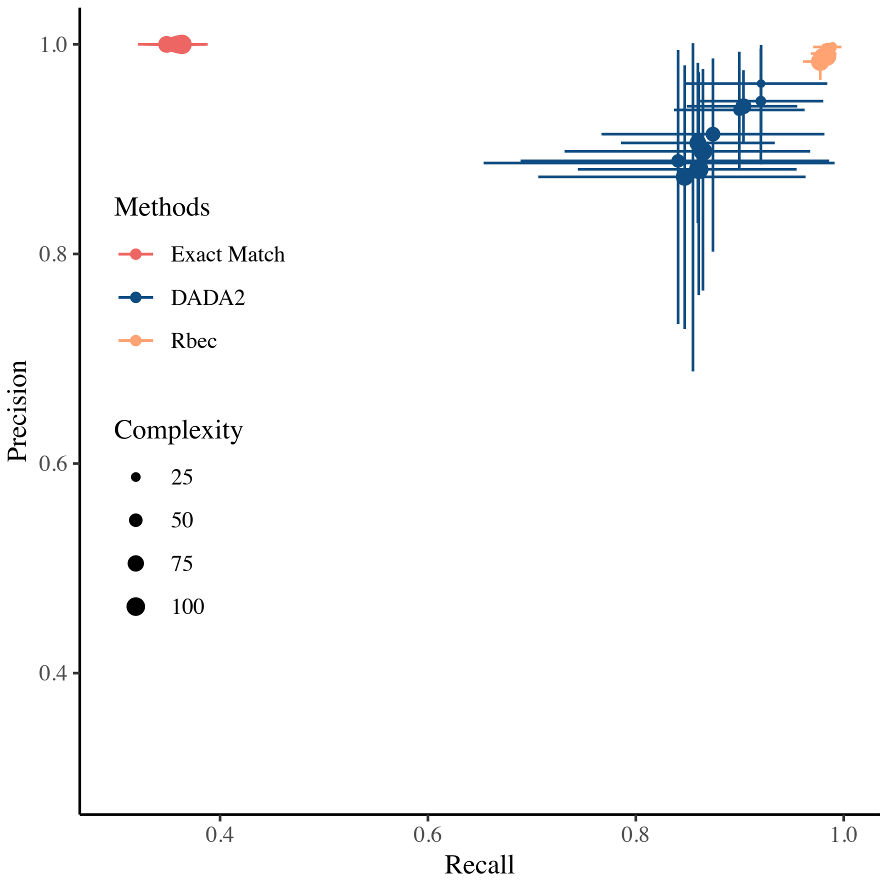

Figure S6. Precision and recall of different methods. Bacterial mock communities with different complexities were analysed to calculate the precision and recall of different methods. We used the formulas $Recall = \frac{No.\ of\ precisely\ corrected\ \text{reads}}{Total\ No.\ of\ reads}$ and $Precision = \frac{No.of\ precisely\ corrected\ reads}{No.of\ corrected\ reads}$. Crossed vertical and horizontal lines represent the standard deviation of precision and recall in each complexity threshold, while dots indicate the mean values.

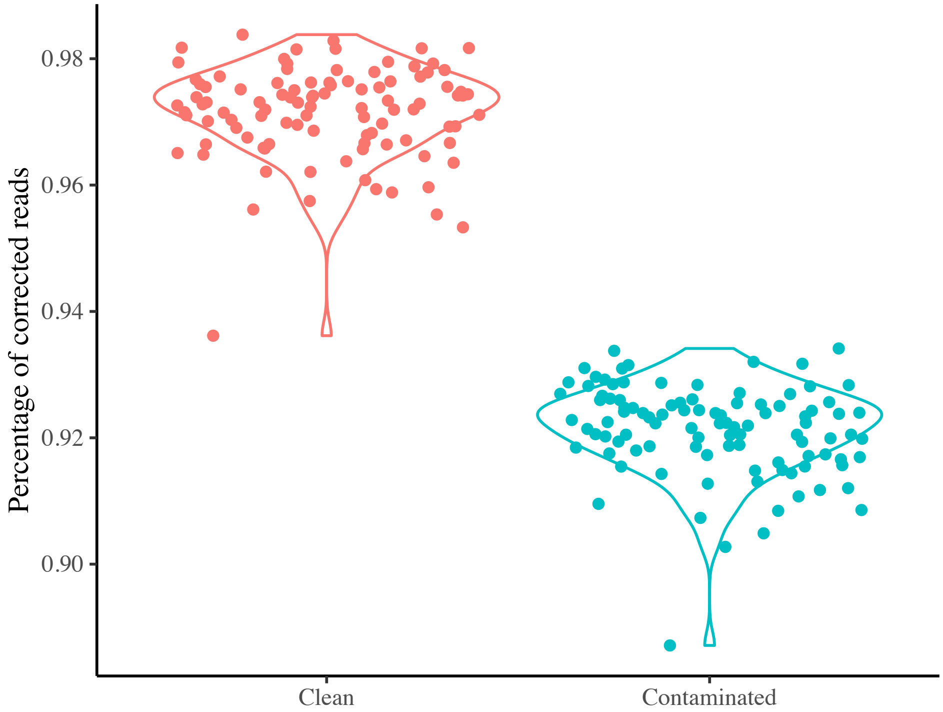

Figure S7. Segregation of percentages of corrected reads between clean and contaminated SynCom samples. A set of 100 'clean' mock communities were generated by randomly picking up 50 bacteria from the bacterial seed pool and mixing the subsampled reads from corresponding strains. To generate a comparable set of contaminated samples, amplicon reads from the *E. coli* K12 *16S* rRNA sequence were added to each clean mock community to make up 5% relative abundance.

Supplementary Tables

Method	CPU Time (s)
AmpliCI	277(±14.2)
Closed picking	10(±0.3)
DADA2	126.1(±6.1)
Deblur	370.6(±8.6)
Exact match	203.3(±1.7)
Rbec	126.1(±4.5)
Unoise	0.4(±0)

Table S1. Time performance of different methods measured by CPU time (in seconds) for different methods tested using 5 SynComs samples with 10 000 reads in each on single CPU.

References

1. Callahan BJ, McMurdie PJ, Rosen MJ, Han AW, Johnson AJA, Holmes SP. DADA2: High-resolution sample inference from Illumina amplicon data. *Nat Methods* 2016; **13**: 581–583.

2. Shen W, Le S, Li Y, Hu F. SeqKit: A Cross-Platform and Ultrafast Toolkit for FASTA/Q File Manipulation. *PLoS One* 2016; **11**: e0163962.

3. Magoc T, Salzberg SL. FLASH: fast length adjustment of short reads to improve genome assemblies. *Bioinformatics* 2011; **27**: 2957–2963.

4. Edgar RC. Search and clustering orders of magnitude faster than BLAST. *Bioinformatics* 2010; **26**: 2460–2461.

5. Bolyen E, Rideout JR, Dillon MR, Bokulich NA, Abnet CC, Al-Ghalith GA, et al. Reproducible, interactive, scalable and extensible microbiome data science using QIIME 2. *Nat Biotechnol* 2019; **37**: 852–857.

6. Peng X, Dorman KS. AmpliCI: a high-resolution model-based approach for denoising Illumina amplicon data. *Bioinformatics* 2020.

7. Edgar RC, Flyvbjerg H. Error filtering, pair assembly and error correction for next-generation sequencing reads. *Bioinformatics* 2015; **31**: 3476–3482.

8. Oksanen AJ, Blanchet FG, Friendly M, Kindt R, Legendre P, Mcglinn D, et al. vegan: Community Ecology Package. *R Packag version 25-7* 2020.

9. Bai Y, Müller DB, Srinivas G, Garrido-Oter R, Potthoff E, Rott M, et al. Functional overlap of the Arabidopsis leaf and root microbiota. *Nature* 2015; **528**: 364–369.

10. Durán P, Thiergart T, Garrido-Oter R, Agler M, Kemen E, Schulze-Lefert P, et al. Microbial Interkingdom Interactions in Roots Promote Arabidopsis Survival. *Cell* 2018; **175**: 973-983.e14.

11. Guo X, Zhang X, Qin Y, Liu Y-X, Zhang J, Zhang N, et al. Host-Associated Quantitative Abundance Profiling Reveals the Microbial Load Variation of Root Microbiome. *Plant Commun* 2020; **1**: 100003.
